# Opposition between PKC isoforms regulates histone deimination and neutrophil extracellular chromatin release

**DOI:** 10.3389/fimmu.2013.00038

**Published:** 2013-02-20

**Authors:** Indira Neeli, Marko Radic

**Affiliations:** Department of Microbiology, Immunology and Biochemistry, University of Tennessee Health Science CenterMemphis, TN, USA

**Keywords:** NETosis, PAD4, protein kinase C, deimination, inflammation

## Abstract

In response to inflammation, neutrophils deiminate histones and externalize chromatin. Neutrophil extracellular traps (NETs) are an innate immune defense mechanism, yet NETs also may aggravate chronic inflammatory and autoimmune disorders. Activation of peptidylarginine deiminase 4 (PAD4) is associated with NET release (NETosis) but the precise mechanisms of PAD4 regulation are unknown. We observed that, in human neutrophils, calcium ionophore induced histone deimination, whereas phorbol myristate acetate (PMA), an activator of protein kinase C (PKC), suppressed ionophore-induced deimination. Conversely, low doses of chelerythrine and sanguinarine, two inhibitors of PKC, reversed PMA inhibition and enhanced ionophore-stimulated deimination. In addition, a peptide inhibitor of PKCα superinduced ionophore activation of PAD4, thus identifying PKCα as the PMA-induced inhibitor of PAD4. At higher doses, chelerythrine, sanguinarine, and structurally unrelated PKC inhibitors blocked histone deimination, suggesting that a different PKC isoform activates histone deimination. We identify PKCζ as activator of PAD4 because a specific peptide inhibitor of this PKC isoform suppressed histone deimination. Confocal microscopy confirmed that, in the presence of PMA, NETosis proceeds without detectable histone deimination, and that ionophore cooperates with PMA to induce more extensive NET release. Broad inhibition of PKC by chelerythrine or specific inhibition of PKCζ suppressed NETosis. Our observations thus reveal an intricate antagonism between PKC isoforms in the regulation of histone deimination, identify a dominant role for PKCα in the repression of histone deimination, and assign essential functions to PKCζ in the activation of PAD4 and the execution of NETosis. The precise balance between opposing PKC isoforms in the regulation of NETosis affirms the idea that NET release underlies specific and vitally important evolutionary selection pressures.

## INTRODUCTION

Neutrophils are the first responders of the innate immune system (Nathan, 2006). By their rapid and effective response to infections, neutrophils perform an essential role in the defense of the host from various pathogens. Neutrophils circulate in the blood which carries them to the site of an infection. Within seconds, they adhere to the activated endothelium and pass through or between endothelial cells into tissues (Phillipson et al., 2006). There, neutrophils migrate along gradients of inflammatory mediators toward the ongoing infection. Upon arrival, they engage bacteria, viruses, or fungi through alternative and complementary mechanisms, including phagocytosis, production of reactive oxygen, and the secretion of bactericidal substances (Kennedy and DeLeo, 2009). Despite the vital role of neutrophil responses to infections, we have only an incomplete understanding of neutrophil functions. The study of neutrophils *ex vivo* is complicated by their short lifetime and the difficulty of recreating characteristics of an infection in a culture dish. Pharmacological stimuli that are capable of eliciting neutrophil activation *in vitro* are therefore widely used in the analysis of neutrophil responses to infections.

Phorbol-12-myristate-13-acetate (PMA) is useful for its ability to activate neutrophils and elicit responses that include enhanced adhesion, production of reactive oxygen, and degranulation (Tauber, 1987). PMA elicits these responses because it can penetrate the cell membrane and mimic diacylglycerol (DAG), a cellular signal that activates two of the three families of protein kinase C (PKC). The PMA-responsive PKC belong to the classical (α, β, γ) and novel (δ, υ, τ) PKC families, whereas the alternative PKC isoforms (ζ, λ/ι) function independent of DAG/PMA (Steinberg, 2008). The classical PKC are distinguished from all other PKC because they also require elevated calcium for maximal activity. The compounds A23187 and ionomycin serve as ionophores that form channels in the plasma membrane and allow influx of calcium ions (Erdahl et al., 1994). In many studies, ionophores are used in combination with PMA to elicit maximal activation of cellular responses that require elevated intracellular calcium and PKC activation.

Phorbol myristate acetate and ionophore are strong stimuli for multiple pathways in neutrophils. Thus, a plausible prediction was that PMA or ionophore would affect the manner in which neutrophils die. PMA induces a unique cell death that differs from apoptosis and necrosis (Takei et al., 1996; Suzuki and Namiki, 1998). The discovery that this novel form of neutrophil cell death involves the release of nuclear chromatin and serves in the innate neutrophil response to pathogens (Brinkmann et al., 2004) led to a fundamental paradigm shift in our thinking about innate immune responses to infections. Indeed, the cell death that is induced by PMA is in many ways analogous to the response of neutrophils to bacterial, fungal, protozoan, and viral pathogens (Brinkmann and Zychlinsky, 2012). Thus, PMA found a new use in studies on the mechanisms of neutrophil innate responses to pathogens.

When exposed to pathogens, or to particulates in the presence of inflammatory stimuli, neutrophils decondense chromatin, and rupture the nuclear envelope which allows the nuclear chromatin to expand into the cytoplasm (Fuchs et al., 2007). In parallel, cytoplasmic granules burst, releasing bactericidal contents into the cytoplasm where they associate in a tight complex with the unraveling chromatin. The last and most dramatic step in this programed cell death pathway is the release of chromatin to the extracellular space. The externalized chromatin can immobilize pathogens and is therefore referred to as a neutrophil extracellular trap (NET). The cell death resulting in NET chromatin release is called NETosis (Steinberg and Grinstein, 2007). A variant form of NETs is composed of mitochondrial DNA and their release may not lead to cell death (Yousefi et al., 2009). Indeed, *in vivo* observations in mice infected by *S. aureus* indicate that release of nuclear chromatin NETs may not induce the immediate loss of cellular functions (Yipp et al., 2012), although functions that depend on gene expression are presumably compromised in these neutrophils.

The release of chromatin follows the activation of peptidylarginine deiminase 4 (PAD4), an enzyme that converts arginine residues to citrullines (Neeli et al., 2008; Wang et al., 2009). PAD4 is activated by inflammatory stimuli and bacterial breakdown products (Neeli et al., 2008). Its activation depends on signals from the cell surface and on an intact cytoskeleton (Neeli et al., 2009). Of the five mammalian deiminases, PAD4 is the enzyme that localizes to the nucleus and modifies histones (Nakashima et al., 2002). Because mutant mouse neutrophils that lack PAD4 cannot release NETs (Li et al., 2010; Hemmers et al., 2011), it has been argued that NET release depends on the activity of PAD4. Just how PAD4 mediates NET release is not known. One possibility is that deimination serves a structural role in the global relaxation of chromatin that precedes NET release: deimination reduces the positive charge of histones which may unravel the tightly packed nuclear chromatin and promote NETosis (Wang et al., 2009). Alternatively, deimination could reprogram gene expression in analogy with other post-translational modification (PTM) and thus set the stage for the execution of NETosis in response to inflammation (Cuthbert et al., 2004; Wang et al., 2004). To distinguish between these possibilities, we must learn more about the regulation of PAD4 in neutrophils. Here, we report consequences of PKC isoform activation and repression on histone deimination and NETosis.

## MATERIALS AND METHODS

### ANTIBODIES AND CHEMICALS

We obtained the rabbit antibody (Ab) to the carboxy terminus of histone H3 and the Ab to modified citrulline from Millipore (Temecula, CA, USA). The rabbit Ab to deiminated histone H3 (dH3) was from Abcam (ab 5103). The horseradish peroxidase (HRP)-conjugated secondary Abs to rabbit immunoglobulin G (IgG; A0545), goat anti-rabbit conjugated to AF488, calcium ionophore (A23187), ionomycin (I0634), PMA (P8139), and protease inhibitor cocktail (P8340) were purchased from Sigma-Aldrich. Sytox orange was obtained from Invitrogen Life Technologies. All enzyme inhibitors used in this paper were purchased from Calbiochem (Millipore).

### HUMAN NEUTROPHIL ISOLATION

Neutrophils were isolated from buffy coats obtained from healthy donors (Lifeblood Biological Services, Memphis, TN, USA) in accord with protocols approved by the University of Tennessee Institutional Review Board and isolated following published methods (Neeli et al., 2008). Briefly, neutrophils were purified at room temperature (RT), enriched in the supernatant of an Isolymph sedimentation and recovered in the pellet of an Isolymph density gradient (Gallard Schlesinger) under endotoxin-free conditions. The contaminating erythrocytes were lysed in ice-cold hypotonic (0.2%) sodium chloride solution for 30 s, at which point the solution was rendered physiologic saline by addition of hypertonic (1.6%) sodium chloride. The neutrophils were rinsed once in Hanks’ balanced salt solution (HBSS; without calcium or magnesium) and re-suspended at 2 × 10^6^ cells/ml. At that point, neutrophil viability was assessed by trypan blue dye exclusion and exceeded 98%. Neutrophils were kept at RT (for up to 30 min) before use in experiments.

### TREATMENTS OF NEUTROPHILS AND DETERMINATION OF HISTONE DEIMINATION

One million cells in 500 μl of HBSS were incubated in polypropylene tubes with or without different inhibitors for 30 min at 37°C. Then, 2× stimuli were added in 500 μl of HBSS containing 200 μM calcium. For lipopolysaccharide (LPS) stimulation, we added 1% human serum as a source of LPS-binding protein to phosphate buffered saline (PBS) containing 100 μM calcium and 100 ng/ml LPS. Following 2 h of incubation, the cells were centrifuged at 1200 rpm for 5 min, the buffer was discarded and the cell pellet was lysed by adding 50 μl of sodium dodecyl sulfate (SDS) lysis buffer. Samples were boiled for 5 min, cooled and stored at -20°C until use.

### PREPARATION AND ANALYSIS OF NETs

To prepare NETs, 1 × 10^6^ purified neutrophils were plated in 500 μl of serum-free HBSS containing 100 μM CaCl_2_ per well in a 24-well tissue culture plate. Ionophore, PMA or a combination of the two compounds were added as stimuli to induce NETosis. At the end of 1, 2, or 4 h incubations, 0.5 U of micrococcal nuclease (Worthington) was added to the wells and the plate was incubated for another 10 min at 37°C. Supernatants were collected and adjusted to 2 mM ethylenediaminetetraacetic acid (EDTA), clarified by centrifugation at 5,000 *g* for 5 min, and saved for analysis of DNA and myeloperoxidase (MPO). DNA concentrations were determined by Pico-Green fluorescence (“Quant-It,” Life Technologies). MPO activity was measured in parallel aliquots by oxidation of tetramethylbenzidine (TMB, 2 mM) in the presence of H_2_O_2_, followed by spectrophotometry.

### WESTERN BLOTTING

Cell lysates were resolved by 12% SDS-PAGE (polyacrylamide gel electrophoresis), and blotted to nitrocellulose membranes. Membranes were blocked for 1 h at RT with 5% bovine serum albumin (BSA) or 5% milk in TBST [Tris-buffered saline (TBS) and Tween 20, 25 mM Tris (pH 7.2), 150 mM NaCl, and 0.1% Tween 20] and rinsed before overnight incubation at 4°C with a dilution of primary Abs in TBST. Subsequently, membranes were washed and incubated for 1 h with goat anti-rabbit Ab conjugated to HRP, washed three times with TBST and twice with TBS alone. The HRP activity was detected by using chemiluminescence reagent plus (PerkinElmer Life Sciences) and exposure to autoradiographic film. For detection of citrullines, membranes were blocked with 1% ovalbumin and incubated with chemical reagents that modify citrulline by formation of an ureido group, as suggested by the manufacturer.

### CONFOCAL MICROSCOPY

Neutrophils were allowed to settle for 30 min at 37°C onto glass coverslips that were precoated with poly-L-lysine. The cells were treated with stimuli in the presence or absence of various inhibitors (or left untreated) and incubated for 2 h at 37°C. The coverslips were washed with ice-cold HBSS, the cells were fixed with 4% paraformaldehyde in HBSS and blocked 1 hr at RT with blocking solution (HBSS with 1% BSA, 0.05% Tween 20, and 2 mM EDTA). The coverslips were washed with wash buffer (HBSS with 1% BSA), incubated with rabbit anti-citrullinated histone H3 Abs (diluted 1/100 in wash buffer), washed again, incubated with goat anti-rabbit IgG coupled with AF488 together with Sytox orange for 30 min at RT, and analyzed by confocal microscopy, as previously described. Viability of neutrophils exposed to various inhibitors was assessed by trypan blue exclusion following a 2-h incubation. Cell viability ranged between 90 and 95% for any of the inhibitors at the highest concentrations used in our assays. The exception was calphostin C that at 100 μM reduced viability of treated cells to 80%.

## RESULTS

### DISTINCT EFFECTS OF NEUTROPHIL ACTIVATORS ON HISTONE DEIMINATION AND NET RELEASE

**FIGURE 1:**
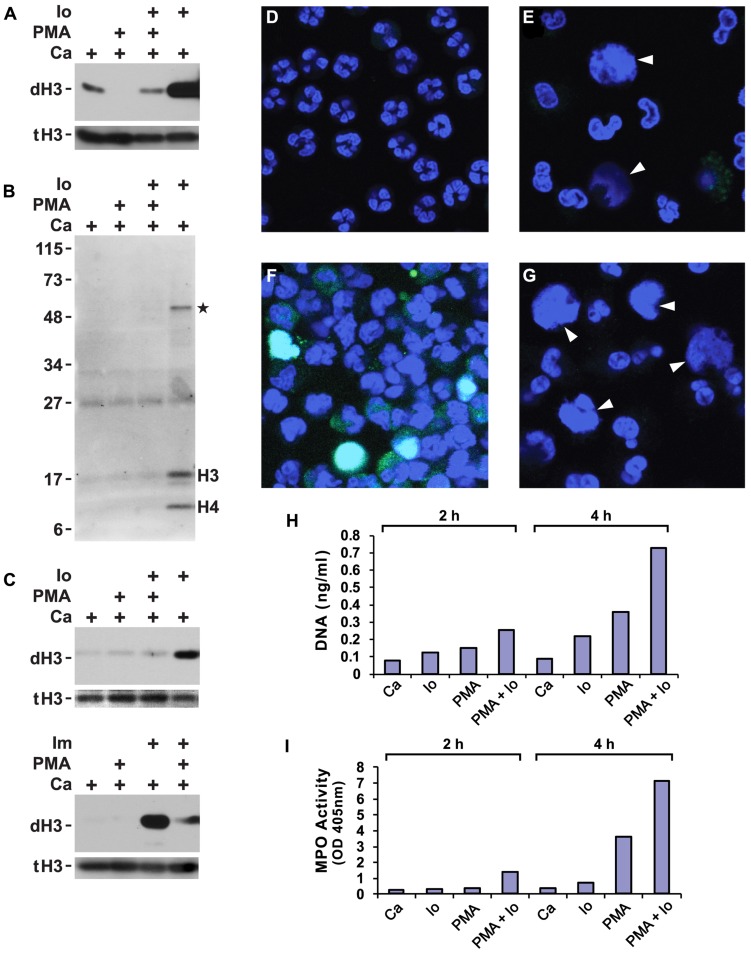
**Phorbol myristate acetate (PMA) suppresses histone deimination, whereas ionophore or ionomycin stimulate deimination**. Purified neutrophils were incubated in buffers containing calcium (Ca), A23187 ionophore (Io) or ionomycin (Im), and/or PMA for 2 h. Cells were lysed and analyzed by western blotting to detect deiminated histone H3 (dH3) vs. total H3 (tH3). The presence of stimuli is indicated by a plus sign. Panels **(A,B)** show blots of lysates from cells incubated with the indicated stimuli or calcium alone. Panel **(A)** shows results of a blot detecting dH3, whereas panel **(B)** shows antibody binding to modified citrullines. Migration of H3 and H4 histones and position of molecular weight markers are indicated. Asterisk indicates a protein of approximately 50 kD that is deiminated in response to ionophore treatment. Substitution of PBS for HBSS did not qualitatively alter the results of the treatments (**C**, top), nor did substitution of ionomycin for ionophore (**C**, bottom). The highest levels of dH3 were observed after ionophore or ionomycin treatment. Inclusion of PMA reduced histone deimination and PMA alone failed to stimulate deimination. Microscopy of unstimulated neutrophils showed cells with multilobed nuclei **(D)**, whereas PMA treatment induced nuclear swelling, rupture, and NET release **(E)**. In these pictures, DNA is displayed in blue and binding of antibodies to deiminated histone H3 in green. Diffuse NETs are indicated by arrow heads here and in **(G)**. Ionophore induced histone deimination in cytoplasm and decondensed nuclei **(F)**. A combination of PMA and ionophore led to enhanced NETosis with little to no detectable histone deimination **(G)**. Purified NET DNA was solubilized by micrococcal nuclease digestion and quantified by fluorescence **(H)**. MPO released by nuclease treatment was measured in solution by the TMB method **(I)**. All experiments were performed at least five times with consistent results.

A wide variety of inflammatory stimuli induce histone deimination and NET release (Rohrbach et al., 2012). Quite commonly, PMA or the A23187 ionophore have been used as stimuli for NETosis. Hence, we wished to determine the extent of histone deimination following administration of these compounds to purified human neutrophils. We also set out to determine whether the two stimuli can be used in combination. In buffers that contained 100 μM calcium, ionophore strongly induced histone deimination (**Figure 1A**). In contrast, PMA did not increase histone deimination above background. Instead, PMA diminished the low level of histone deimination that was induced by calcium alone. Strikingly, PMA reduced ionophore-induced deimination to background levels when the two were added simultaneously to neutrophils. To exclude the possibility that PMA induces a citrulline distribution in histone H3 that escapes detection by the anti-citrullinated H3 reagent, we probed a duplicate blot with an Ab that recognizes modified citrullines (**Figure 1B**). This reagent confirmed that PMA treatment does not induce deimination of any proteins in the cell and that ionophore treatment results in the deimination of histones H3 and H4 as the predominant PAD4 substrates. At 2 h after PMA addition, one additional band was detected at approximately 50 kD (**Figure 1B**). This band may correspond to the previously identified PAD2 substrate actin (Darrah et al., 2012). We must insert a cautionary note here: certain modifications may remain below the detection limit of the reagents we used to detect modified citrullines. However, it is valid to point out that the reagents clearly detect citrulline in lysates from ionophore treated neutrophils. Thus, PMA does not induce histone deimination that is detected by histone-specific or by modified citrulline-specific antibodies.

Suppression of deimination by PMA was observed in different physiological buffers and with different calcium ionophores. In PBS or HBSS (each supplemented to 100 μM calcium), the A23187 ionophore induced histone deimination that was inhibited to background levels by PMA (**Figure 1C**). Substitution of ionomycin for A23187 (in HBSS buffer) induced deimination that was greatly diminished by addition of PMA (**Figure 1C**). These results indicate that an increase in cytoplasmic calcium stimulates histone deimination by PAD4 but that a target of PMA, presumably a PMA-dependent PKC isoform, acts in a dominant fashion to suppress deimination.

Because most stimuli for histone deimination also induce NETosis (Neeli et al., 2009), we decided to test whether PMA and ionophore, separately or combined, induce NETosis. Untreated, freshly isolated neutrophils displayed multilobed nuclei of uniform size (**Figure 1D**). Upon incubation with PMA, nuclei swelled and constrictions between the nuclear lobes widened. Some nuclei released their chromatin into the cytoplasm and other cells dispersed NETs (**Figure 1E**). In agreement with western blot results, NETs induced by PMA contained very little or no dH3. The NETs shown here (indicated by arrow heads in **Figures 1E,G**) resemble the diffuse and cloud-like structures seen by live-cell microscopy (Fuchs et al., 2007) or following minimal fixation of cells (Hakkim et al., 2011; Brinkmann and Zychlinsky, 2012), leading us to conclude that NETs acquire the extended, fibrous structure after buffer exchange and cover slip manipulations that are common steps in cell fixation protocols.

A23187 ionophore induced deimination that, in many neutrophils, coincided with nuclear swelling and chromatin release (**Figure 1F**). In contrast, ionophore in combination with PMA readily induced NETosis, yet histone deimination was below our ability to detect (**Figure 1G**). Microscopy thus indicated that A23187 ionophore or PMA, as well as the combination of the two compounds, efficiently induce NETosis, which, in the presence of PMA, proceeds with no detectable histone deimination. The microscopy experiments thus suggested that histone deimination may be dispensable for PMA-induced NETosis.

To quantify NET release induced by different treatments, we solubilized NETs with micrococcal nuclease to measure DNA by fluorescence (**Figure 1H**) and released MPO by enzymatic reaction (**Figure 1I**). Externalized MPO correlates with NET release as most of the MPO is NET-associated (Parker et al., 2012a). In our assays, DNA and MPO measurements concordantly showed that ionophore or PMA induce NETosis but that a combination of the two stimuli is even more effective at eliciting NETosis. These assays confirmed that the two stimuli cooperate in inducing NETosis, whereas PMA counteracts ionophore in the induction of histone deimination.

### PMA INDUCES A PKC ISOFORM THAT SUPPRESSES HISTONE DEIMINATION

**FIGURE 2:**
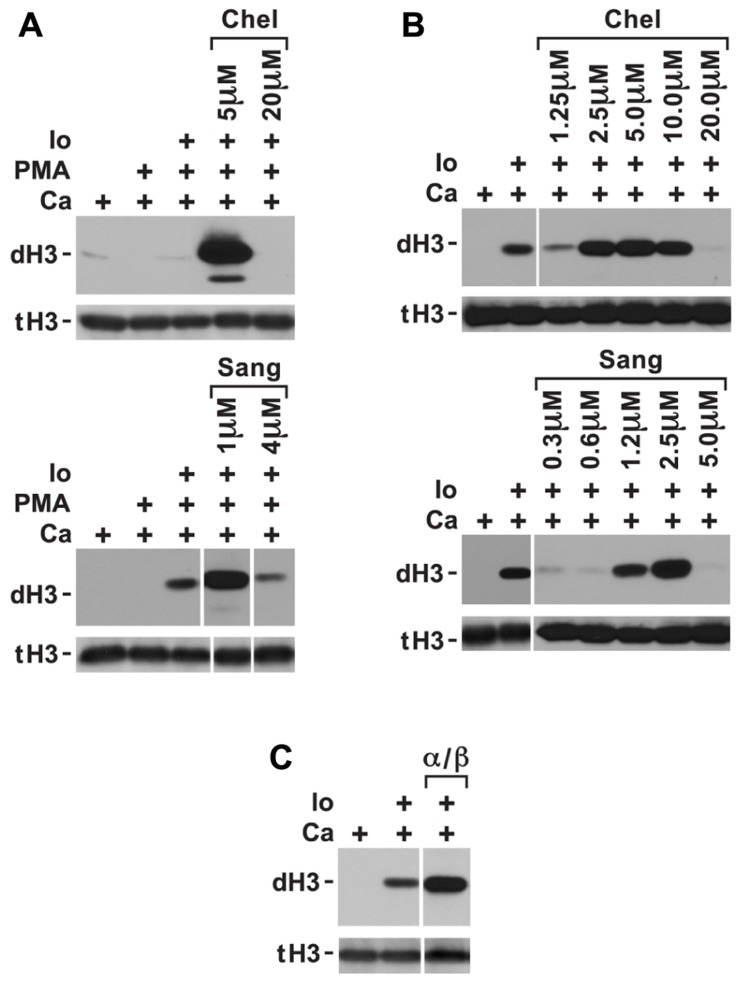
**Conditions in which PKC inhibitors enhance histone deimination**. **(A)** Histone deimination that is repressed by PMA can be derepressed by 5 μM chelerythrine (Chel) or 1 μM sanguinarine (Sang) but higher concentrations of these inhibitors lead to renewed repression. Levels of deiminated histone H3 (dH3) vs. total H3 (tH3) were estimated by western blotting. **(B)** A narrow range of inhibitor concentrations also superinduced histone deimination in response to ionophore (Io). **(C)** Pseudosubstrate peptide inhibitor for PKCα/β enhanced histone deimination that was stimulated by ionophore. The vertical white lines separate lanes from the same gel that were taken with identical autoradiographic exposure but were arranged differently in the original gel. The presence of stimuli is indicated by a plus sign.

Phorbol myristate acetate inhibition of histone deimination may be mediated by a PMA-induced PKC isoform. To test whether PAD4 inhibition could be relieved by repression of the inhibitory PKC, we measured histone deimination in cells treated with PKC inhibitors prior to stimulation with PMA. Indeed, we identified a relatively narrow concentration range of chelerythrine, a plant alkaloid (Powell and Chen, 1955), that could relieve the repression of histone deimination by PMA. To confirm this possibility, we used the related compound sanguinarine (Schenck and Hannse, 1954) and observed that, in the presence of ionophore, 5 μM chelerythrine or 1 μM sanguinarine optimally reversed PMA inhibition (**Figure 2A**). Higher concentrations of the PKC inhibitors (20 μM chelerythrine or 10 μM sanguinarine), however, suppressed deimination. These results suggested that PMA induces, and low concentrations of these PKC inhibitors suppress, a PKC isoform that inhibits histone deimination. Once this isoform is repressed, histone deimination again becomes inducible by calcium ionophore.

To test whether ionophore activates the inhibitory PKC isoform, we added low concentrations of chelerythrine and sanguinarine prior to initiating ionophore treatment of neutrophils. We used this experiment to fine-tune the dose at which chelerythrine and sanguinarine were most effective. Inhibitor concentrations falling between 2.5 and 5.0 μM chelerythrine and near 2.5 μM sanguinarine further enhanced ionophore-induced histone deimination (**Figure 2B**). These results indicated that an elevation of intracellular calcium activates the inhibitory PKC. Because classical PKC isoforms respond to calcium and PMA (Steinberg, 2008), we deduced that the inhibitory PKC isoform is likely a member of the classical group of PKC enzymes. As above, higher levels of the PKC inhibitors, or EDTA added to the medium (data not shown), suppressed deimination.

Because neutrophils express PKCα (Nixon and McPhail, 1999), we repeated the ionophore stimulation in the presence of a specific PKCα/β inhibitor peptide. The myristoylated inhibitor peptide passes the plasma membrane and interacts with the catalytic domain of the classical PKC isoform. The peptide inhibits substrate binding, thus acting as a competitive inhibitor. Addition of the PKCα/β inhibitory peptide increased the extent of histone deimination induced by ionophore (**Figure 2C**), suggesting that PKCα inhibits histone deimination.

### PKCζ IS REQUIRED FOR INDUCTION OF HISTONE DEIMINATION

**FIGURE 3:**
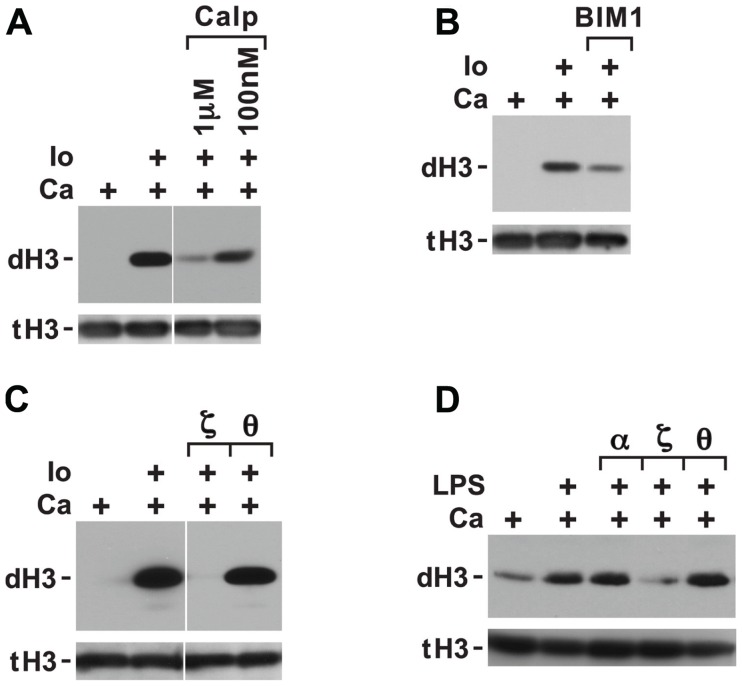
**Histone deimination induced by ionophore is only partially suppressed by calphostin C or bisindolylmaleimide 1 (BIM1) but is fully suppressed by a specific peptide inhibitor of PKCζ**. Calphostin C (Calp) partially suppressed histone deimination **(A)**, as did addition of 100 nM BIM1 **(B)**. These two compounds do not effectively block atypical PKC isoforms. In contrast, ionophore-induced histone deimination was completely suppressed by a peptide inhibitor of PKCζ but not by an inhibitor of PKCτ **(C)**. The vertical white lines separate lanes from the same gel that were taken with identical autoradiographic exposure but were arranged differently in the original gel. The presence of stimuli is indicated by a plus sign. Addition of LPS in PBS (100 ng/ml) enhanced histone deimination, and a peptide inhibitor of PKCζ but not of PKCτ or α/β suppressed the elevated level of deimination **(D)**.

The inhibition of histone deimination by high levels of chelerythrine and sanguinarine (**Figures 2A,B**) suggested that histone deimination is contingent on the activation of a second PKC isoform. This possibility was consistent with our previous observation that treatment of neutrophils with staurosporine, a potent PKC inhibitor, blocks histone deimination (Neeli et al., 2008). To confirm the contribution of a PKC to deimination, we treated neutrophils with calcium ionophore in the presence or absence of additional, broadly effective PKC inhibitors. Pretreatment of neutrophils with calphostin C (**Figure 3A**) or bisindolylmaleimide 1 (BIM1; **Figure 3B**) diminished but failed to completely inhibit the induction of histone deimination by calcium ionophore. Calphostin C and BIM1 are considered relatively ineffective for atypical PKC isoforms (Martiny-Baron et al., 1993; Xu and Clark, 1997).

To pinpoint the PKC isoform that promotes histone deimination, we used peptide inhibitors of PKCτ and PKCζ, two PKC isoforms that have been implicated in neutrophil responses to external stimuli (Laudanna et al., 1998; Bertram et al., 2012). The PKCζ inhibitor completely blocked histone deimination, whereas the PKCτ peptide was ineffective (**Figure 3C**). These results indicated that activation of histone deimination by calcium ionophore depends on the activation of PKCζ.

To test whether PKC isoforms regulate histone deimination induced by stimuli encountered *in vivo*, we incubated neutrophils with specific PKC isoform inhibitors prior to stimulation with LPS (**Figure 3D**). LPS treatment induced histone deimination that could be suppressed by a peptide inhibitor of PKCζ, whereas peptides against PKCα/β or PKCτ were ineffective at suppressing LPS-induced PAD4 activation. This observation suggested that PAD4 activation by PKCζ is a shared signal among diverse neutrophil stimuli.

### EFFECT OF PKC INHIBITORS ON NETosis

**FIGURE 4:**
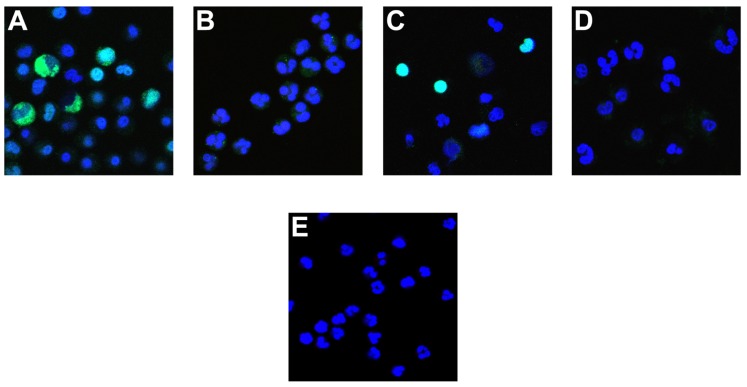
**Effect of PKC inhibitors on NETosis**. Neutrophils were incubated with A23187 ionophore in the presence of 5 μM chelerythrine **(A)**, or 20 μM chelerythrine **(B)**. Antibody binding to deiminated histone H3 (green) and the DNA contained in cells or released as NETs (blue) was observed in 5 μM chelerythrine, whereas 20 μM inhibitor precluded deimination and most other morphologic features of NETosis. Cells incubated with PKCα/β pseudosubstrate peptide inhibitor deiminated histone H3 and proceeded to NET release **(C)**. The PKCζ inhibitor peptide suppressed histone deimination and NET release but earlier stages of NETosis, such as a partial heterochromatin decondensation and nuclear de lobulation, were observed **(D)**. As in **Figure 1E**, untreated neutrophils showed typical multilobed nuclei without apparent evidence of NETosis **(E)**.

To examine the effect of enhanced vs. inhibited histone deimination on the morphology of NETosis, we incubated neutrophils in the presence of various inhibitors prior to stimulation with calcium ionophore. With chelerythine, we observed two different results, depending on the concentration of inhibitor that was used. At 5 μM chelerythrine, neutrophils generated increased levels of deiminated histones and progressed through various stages of NETosis (**Figure 4A**). Interestingly, we detected the highest levels of dH3 in the cytoplasm of neutrophils with decondensing nuclei and the levels remained high in cells with mixed nuclear and cytoplasmic contents. In contrast, neutrophils incubated with 20 μM chelerythrine exhibited drastically reduced histone deimination, and cell morphologies associated with NETosis were largely absent (**Figure 4B**). Therefore, at the lower concentration of chelerythrine, histone deimination and NETosis execution were strongly induced, whereas, at the higher concentration of this PKC inhibitor, both histone deimination and NETosis were largely repressed.

The peptide inhibitors had dramatic effects on histone deimination and NET release. Exposure to the PKCα/β pseudosubstrate inhibitor prior to stimulation by ionophore induced strong histone deimination that was most notable in cells with decondensed and expanded nuclei (**Figure 4C**). Importantly, neutrophils stimulated with ionophore in the presence of PKCα inhibitor, readily completed NETosis. In contrast, neutrophils treated with PKCζ inhibitor showed dramatically decreased deimination and impaired NETosis (**Figure 4D**). We observed early nuclear changes of NETosis, such as nuclei with widened interlobe constrictions, yet the release of NETs was largely repressed and cells resembled neutrophils that were left untreated (**Figure 4E**). Collectively, these results indicate that PKC inhibitors provide versatile tools for the analysis of enzymatic steps that regulate the transitions in nuclear structure during NETosis.

## DISCUSSION

NETosis brings high risks and high rewards for the host (Brinkmann and Zychlinsky, 2012; Kaplan and Radic, 2012). NETs represent an efficient mechanism to immobilize a broad range of pathogens and expose them to a high local concentration of damaging and potentially lethal compounds. Thus, NETs may be a life-saving antimicrobial response. NETs also interface with other granulocyte responses and thus establish an integrated set of defenses against infections. However, the release of NETs along with tissue degrading compounds from granulocytes at the site of an infection has long been recognized as a risky strategy that can damage host tissues (Nathan, 2006). An added risk to the host arises from the contribution of NETs to blood clotting and the formation of thrombi (Fuchs et al., 2010). Therefore, it is vital that the release of NETs be carefully regulated.

One way to regulate NETosis is through regulation of PAD4. PAD4 is intensely induced by inflammation, such that up to one-fifth of all histones in the cell acquire citrullines (Nakashima et al., 2002). Most PAD4 substrate arginines are localized to the flexible histone amino termini that are important for nucleosome stacking and histone DNA interactions. Loss of the positive charges along the histone termini reduces electrostatic interactions with DNA and thus allows chromatin to assume an extended conformation (Leshner et al., 2012). The activation of PAD4 hence may provide the mechanical lever whose mass action drives the major morphologic transformation seen in NETosis. This view is consistent with the severely impaired ability of PAD4-deficient neutrophils to initiate NETosis (Li et al., 2010; Hemmers et al., 2011). Although PAD4 has numerous substrates in the cell, including PAD4 itself (Andrade et al., 2010), modification of core histones may be the major reason for chromatin decondensation and NET release. However, our study indicates that PMA-induced NETosis unfolds in the absence of detectable histone deimination. This finding was unexpected because inflammatory and infection-related stimuli for NETosis that invariably induce both the deimination of histones and the release of NETs. It seems likely that the PMA signal for NETosis is qualitatively different from more physiologic stimuli (Parker et al., 2012b). Nevertheless, it is remarkable that a NET stimulus as potent as PMA induces NETs without apparent activation of PAD4. Thus, it is important to more clearly understand the regulation and consequences of PAD4 activation in the induction of NETosis.

Many authors have proposed that PAD4 activation results from an increase in intracellular calcium concentration. This passive model of PAD4 regulation implies that any spike in intracellular calcium, such as may result from a break in the membrane barrier of neutrophils, would activate PAD4. The model is consistent with the strong activation of PAD4 by ionophore and ionomycin (**Figure 1C**). However, our results demonstrate that PAD4 activation is not a direct outcome of elevated levels of intracellular calcium. Even in the presence of elevated cytoplasmic calcium, PMA was capable of repressing deimination. This suggests that neutrophils possess a PMA-responsive mechanism that inhibits PAD4 activation. Under physiologic conditions, this PKC-dependent pathway may prevent the premature activation of PAD4. A PKC isoform that blocks deimination is induced by PMA and calcium and is suppressed by a narrow concentration range of chelerythrine and sanguinarine. Because a specific peptide inhibitor of PKCα/β could overcome PMA repression of histone deimination, we conclude that PKCα functions as a repressor of histone deimination. PKCα, by its association with adhesion receptors (Rucci et al., 2005), may inhibit PAD4 activation early during the attachment of neutrophils to the endothelium and may release PAD4 from repression only during later stages of chemotactic migration.

The most striking finding of our study is that repression by a PKC isoform is not the only safeguard preventing histone deimination by PAD4. In addition, a second PKC isoform is required to induce histone deimination in response to PMA or LPS. Because the induction of deimination was prevented by a specific peptide inhibitor of PKCζ, we conclude that histone deimination requires activation through PKCζ. PKCζ is a conserved, atypical isoform of PKC that contributes to various functions in metazoans, ranging from cell polarity and migration to innate responses to infection (Xu and Clark, 1997; Laudanna et al., 1998; Feng and Longmore, 2005; Nishimura et al., 2005). The remarkable range of PKCζ regulated functions reflects its multiple cellular locations and interacting partners. In infections, PKCζ mediates functions of Toll-like receptors (TLR) at the cell surface (Yang et al., 2011), the assembly of a functional nicotinamide adenine dinucleotide phosphate (NADPH) oxidase complex (Dang et al., 2001; Raad et al., 2009), the activation of latent cytoplasmic nuclear factor-kappaB (NF-κB) subunits (Duran et al., 2003), and their association with active chromatin domains (Levy et al., 2011). From these known PKCζ functions, it follows that PKCζ may have diverse opportunities to regulate histone deimination.

The PKCζ isoform could activate PAD4 as soon as a suitable stimulus binds a cell surface receptor. The ζ isoform participates in the response to *N*-formyl-methionyl-leucyl-phenylalanine (fMLP), adhesion, and chemotaxis (Laudanna et al., 1998). Alternatively, PAD4 could be activated indirectly, following PKCζ-assisted assembly of an active NADPH oxidase complex (Raad et al., 2009). This possibility may link PAD4 activation to the generation of reactive oxygen. A third possibility is that PKCζ regulates the translocation of PAD4 into the nucleus, by analogy to the regulation of LKB1 nuclear localization by PKCζ (Xie et al., 2009). Even though cytoplasmic and nuclear pools of PAD4 may coexist, it is unknown whether PAD4 redistributes following stimulation. A fourth possibility is that PKCζ regulates PAD4 activity by altering the structure of chromatin or its interactions with enzymes that establish the histone epigenetic code (Wang et al., 2010). These alternatives are now amenable to experimentation.

It is remarkable that histone deimination is regulated by two PKC isoforms. This indicates that granulocytes precisely control PAD4 activation and that NETosis depends on a specific set of circumstances. Our results further emphasize that evolutionary pressure ensures histone deimination is closely regulated by an ancient family of kinases that coordinate numerous other cellular responses to environmental conditions. Thus, it is no longer conceivable that NETosis is a random or accidental event. Rather, NETosis is a fundamental response to a challenge to the survival of the organism.

## Conflict of Interest Statement

The authors declare that the research was conducted in the absence of any commercial or financial relationships that could be construed as a potential conflict of interest.
